# *Quisqualis indica* extract ameliorates low urinary tract symptoms in testosterone propionate-induced benign prostatic hyperplasia rats

**DOI:** 10.1186/s42826-020-00059-9

**Published:** 2020-08-08

**Authors:** Dae-geon Kim, Hyo-Jeong Kwon, Jong-Hwan Lim, Joo-heon Kim, Kyu Pil Lee

**Affiliations:** 1 Department of Veterinary Physiology, College of Veterinary Medicine, Gyeongsang National University, Jinju, 52828 Republic of Korea; 2 Department of Veterinary Pathology, College of Veterinary Medicine, Chungnam National University, Daejeon, 34134 Republic of Korea; 3 Huons Research Center, Ansan, Gyeonggi-do 15588 Republic of Korea; 4 Institute of Animal Medicine, College of Veterinary Medicine, Gyeongsang National University, Jinju, 52828 Republic of Korea; 5 Department of Veterinary Physiology, College of Veterinary Medicine, Chungnam National University, Daejeon, 34134 Republic of Korea

**Keywords:** Benign prostatic hyperplasia, *Quisqualis indica*, Intraurethral pressure, Lower urinary tract symptoms

## Abstract

Benign prostate hyperplasia (BPH) is a common disease in old-age males, accounting for approximately 77% of morbidity within the age range of 40 to 70 years. It has been shown that morbidity increases with social graying. *Quisqualis indica linn* (QI) has been used to treat inflammation, stomach pain, and digestion problems. In this study, we evaluated the symptom-regulating effects of QI extract on a testosterone-induced BPH rat model. After inducing BPH in rats using testosterone propionate (TP) injection, we assessed basal intraurethral pressure (IUP) and increments of IUP elicited by electrical field stimulation (5 V, 5, 10, or 20 Hz) or phenylephrine (Phe) (0.01, 0.03, 0.1 mg/kg IV). To induce BPH, 8-week-old rats were subjected to a daily subcutaneous TP (3 mg/kg) injection for 4 weeks. Finasteride (Fina) (10 mg/kg PO) was administered to the rats in the first treatment, while QI (150 mg/kg PO) was administered to those in the second group. Blood pressure was measured together with IUP, after which low urinary tract (LUT), ventral prostate (VP), testicle, and corpus spongiosum were isolated and weighed. Basal IUPs for the Fina- and QI-treated groups were 87.6 and 86.8%, respectively.

LUT and VP organ weights in the QI group were lower than those in the Fina group. However, the QI group showed significantly reduced electrical stimulated or Phe-induced IUP increment compared to the Fina and BPH groups. These results proved that QI can be beneficial for BPH symptoms by inhibiting 5α-reductase and consequently decreasing prostate and releasing urinary pressure.

## Introduction

Benign Prostate Hyperplasia (BPH) is a common disease in old-age males that accounts for 77% of morbidity within the age range of 40 to 70 years [1]. It has been shown that morbidity increases with social graying [2]. Men with BPH can experience great discomfort with urination due to urethral obstruction and irritation of the bladder. Obstructive symptoms include decreased voiding power, intermittent urine and delayed urination. Bladder irritation symptoms include frequent urination and nocturnal urination [3]. Although the pathogenesis of BPH is not fully understood, dihydrotestosterone (DHT) produced by the enzyme 5α-reductase, is known as the main mediator of prostatic stromal and epithelial hyperplasia. Enhanced smooth muscle tone in the urethra, prostate, and bladder neck is also the main contributor to voiding symptoms. Therefore, changes in hormones, including the male hormone, and aging are factors responsible for the enlargement of the prostate gland [4].

Treatment of BPH is mainly through medication. Induction of the relaxation of the prostate smooth muscle by the alpha-adrenergic blocker, restriction of prostate volume using sex hormone suppressors such as 5α-reductase, and relaxation of NO signaling by PDE5 inhibitors are commonly used approaches for BPH [5, 6]. When these medications are ineffective, prostate tissues may be surgically resected. However, less invasive and safer alternative therapies are needed due to surgical complications such as bleeding, erectile dysfunction, and urinary incontinence [7].

Traditionally, *Quisqualis indica linn* (QI) has been used to treat inflammation, stomach pain, and digestion problems [8]. Recently, it has been reported that QI is effective for BPH. It reportedly demonstrated similar efficacy compared to Finasteride at the cellular level, with lesser toxicity at high concentrations [9]. However, no studies have directly measured changes in urethral pressure in response to QI administration in vivo. In this study, we investigated the changes in urethral pressure when QI was administered in testosterone propionate (TP)-induced BPH rats. We also compared the efficacies of QI and finasteride using outcomes from finasteride- and QI-treated rats. We measured IUP in response to hypogastric nerve stimulation or systemic adrenergic stimulation with phenylephrine (Phe) injection to prove the pharmacological efficacy of QI and to provide preclinical evidence.

## Materials and methods

### Preparation of QI extract

The seeds of *Quisqualis indica* (QI) were obtained from a local herbal market in Ansan in Korea, authenticated by Dr. Yeon, and deposited at the herbarium of the HUONS Research Center (Voucher No. HU033/SKJA150427) as described previously [9]. The dried seeds were homogenized to a fine powder (50 kg) and extracted by reflux with 500 L of 70% ethanol at 80 °C for 6 h. The concentration was performed in a vacuum until proper soluble solid-contents were achieved. The concentrated extract was mixed with maltodextrin and subjected to a spray-dryer (ODA-25, SeoGang Engineering, Korea) to obtain the powder from the extract. The standardized QI extract powder contained at least 1% quisqualic acid, as tested by a validated HPLC assay.

### Animals

Seven-week-old male Sprague-Dawley rats were purchased from Koatech (Pyungteck, South Korea). They were maintained under standard laboratory conditions (22 ± 2 °C; relative humidity, 50 ± 5%; 12 h light/dark cycle), and allowed to consume standard rodent chow and sterilized tap water ad libitum. All animal protocols were approved by the Animal Experimental Ethics Committee of Chungnam National University (CNU-01001, Daejeon, South Korea).

### Testosterone propionate-induced BPH and treatments

BPH induction was performed using a modified version of the protocol by Ub Wijerathne, Park et al. [20]. Rats were acclimatized for a week and randomized into 4 groups: (a) normal control (CTRL) group (oral PBS and subcutaneous injection of corn oil); (b) BPH group (BPH) (oral PBS and subcutaneous injection of TP [3 mg/kg body weight (BW); Tokyo Chemical Ins. Co.]; (c) positive control group (Fina) (oral finasteride [10 mg/kg BW; Sigma, St. Louis, MO, U.S.A.] and subcutaneous injection of TP [3 mg/kg BW]); and (d) QI group (QI) (oral QI [150 mg/kg BW] and subcutaneous injection of TP [3 mg/kg BW]). All rats received treatments once per day for 4 weeks.

### Measurement of intraurethral pressure

After BPH was induced and all treatments were provided, intraurethral pressure (IUP) was measured using modified versions of previously described methods [5, 10–12]. After the rats were anesthetized with a combination of urethane (1000 mg/kg, IP) and alpha-chloralose (50 mg/kg, IP), the bladder was exposed through a midline incision in the abdomen. The bladder was cut through the dome and a polyethylene tube (SP45; Natsume, Tokyo, Japan) was inserted towards the bladder neck. A tube with suture was pressed against the outside of the bladder wall near the bladder outlet. Normal saline was continuously infused into the intraurethral lumen through the tube at a constant rate (0.5 mL/10 min) using a NE-4000 syringe pump (New Era Pump Systems, NY, USA). The infusion pressure signals from the urethra were measured using an MLT0699 pressure transducer (AD Instruments, Sydney, NSW, Australia), passed through an amplifier and recorded using a PowerLab 8/35 data acquisition computer system (Software Chart; AD Instruments, Sydney, NSW, Australia). During IUP measurements, the rat was placed on an MP400 warm plate (KITAZATO. Japan) to keep its body temperature at 37 °C. The basal IUP was obtained as an average of 2 min (1 Hz sampling) of the first point of equilibrium.

### Effect of EFS or Phe injection on IUP changes

For the electrical field stimulating the urethra and surrounding prostate tissue, a pair of fine platinum subdermal needles (Grass, RI, USA) was placed in the abdominal cavity. Urethra and prostate tissue were stimulated using an SD9 electrical stimulator (Grass, RI, USA). The pulse duration was 1 ms, and the amplitude was 5 V. Fifty-second trains of pulses were delivered at 5, 10, and 20 Hz at approximately 2-min intervals.

For injection of Phe, a 26-G catheter (BD. Singapore), and heparin cap (BD. Singapore) were placed in the femoral vein. Phe was injected sequentially through the heparin cap at concentrations of 0.01 mg/kg, 0.03 mg/kg, and 0.1 mg/kg. *The changes in IUP after Phe injection* were evaluated as changes in peak (Δpeak) and changes in mean IUP (Δaverage) for time to baseline recovery. All IUP changes were measured using the level before EFS or the injection of Phe was given as the baseline.

### Measurement of blood pressure

The changes in BP, and associated changes in IUP, were evaluated by a direct method. An incision was made on the left or right side of the neck to expose the common carotid artery. The cranial part of the exposed artery was ligated twice with 3–0 silk (Ailee, Busan, Korea), and a 26-G catheter (BD, Singapore) was inserted into the blood vessel. The catheter was connected to an MLT0699 pressure transducer (AD Instruments, Sydney, NSW, Australia) using a polyethylene tube (SP45; Natsume, Tokyo, Japan). This was the same as the measurement of the IUP, and the measured pressure was evaluated by averaging five systolic BP per individual animal.

### Measurement of organ weight

Low urinary tract (LUT), ventral prostate (VP), testicle and corpus spongiosum (CS) were isolated and weighed by autopsy after IUP was measured. All organ weights were presented as percentages of body weight.

### Measurement of CRP

Blood samples (approximately 2 mL) from the cervical artery were collected and placed in a tube containing sodium heparin (15–20 U) during the autopsy. They were centrifuged at 3000 rpm for 5 min to obtain plasma. The obtained plasma was stored at − 80 °C until CRP measurement. Serum CRP was analyzed with a sandwich enzyme-linked immunosorbent assay (ELISA) using a highly sensitive rat sample CRP ELISA kit (LS-F9616, LS bio, USA, Seattle) and microplate absorbance reader (Infinite 200pro, Tecan, Switzerland). The reaction of the rat CRP antibody was evaluated using streptavidin-horseradish peroxidase (HRP). The tetra-methyl benzidine reaction with HRP bound to the immune complex was measured at 450 nm absorbance. The absorbance results were calculated using the four-parameter logistic standard curve fit.

### Isometric contractile recording of ventral prostate tissue ex vivo

Before each experiment, the male SD rats were sacrificed by cervical dislocation following anesthesia using carbon dioxide. The abdominal cavity was immediately opened by midline laparotomy and the urogenital complex was obtained. The urogenital complex was removed from capsular fiber tissues and seminal vesicles. The ventral lobular was excised in strips (approximately 4-mm wide and 10-mm long in ice-cold physiological saline solution (PSS) consisting of 120 mM NaCl, 2.5 mM CaCl_2_, 1.2 mM MgCl_2_, 11 mM glucose, 25 mM NaHCO_3_, 5.9 mM KCl, and 1.2 mM NaH_2_PO_4_.H_2_O. It was also continuously bubbled with 5% CO_2_ and 95% oxygen. The solution was aerated with 95% oxygen and 5% CO2, and warmed 37 °C at pH 7.4. One side of the tissue was connected to the holder, and the other end was connected to an isometric force transducer (FT03, Grass). The collected signal was amplified and converted to a digital signal using a Powerlab system (Powerlab 8/30. ADI instrument, USA). Finally, the collected signal was recorded using the LabChart 8.0 (ADinstrument) program.

### Statistical analysis

All data are expressed as the mean ± standard error of the mean (S.E.M.). Data were analyzed by one-way ANOVA using Origin 8.0 software (OriginLab, MA, USA). A *p*-value below 0.05 was considered statistically significant.

## Results

### Treatment effects of IUP baseline changes

**Fig. 1:**
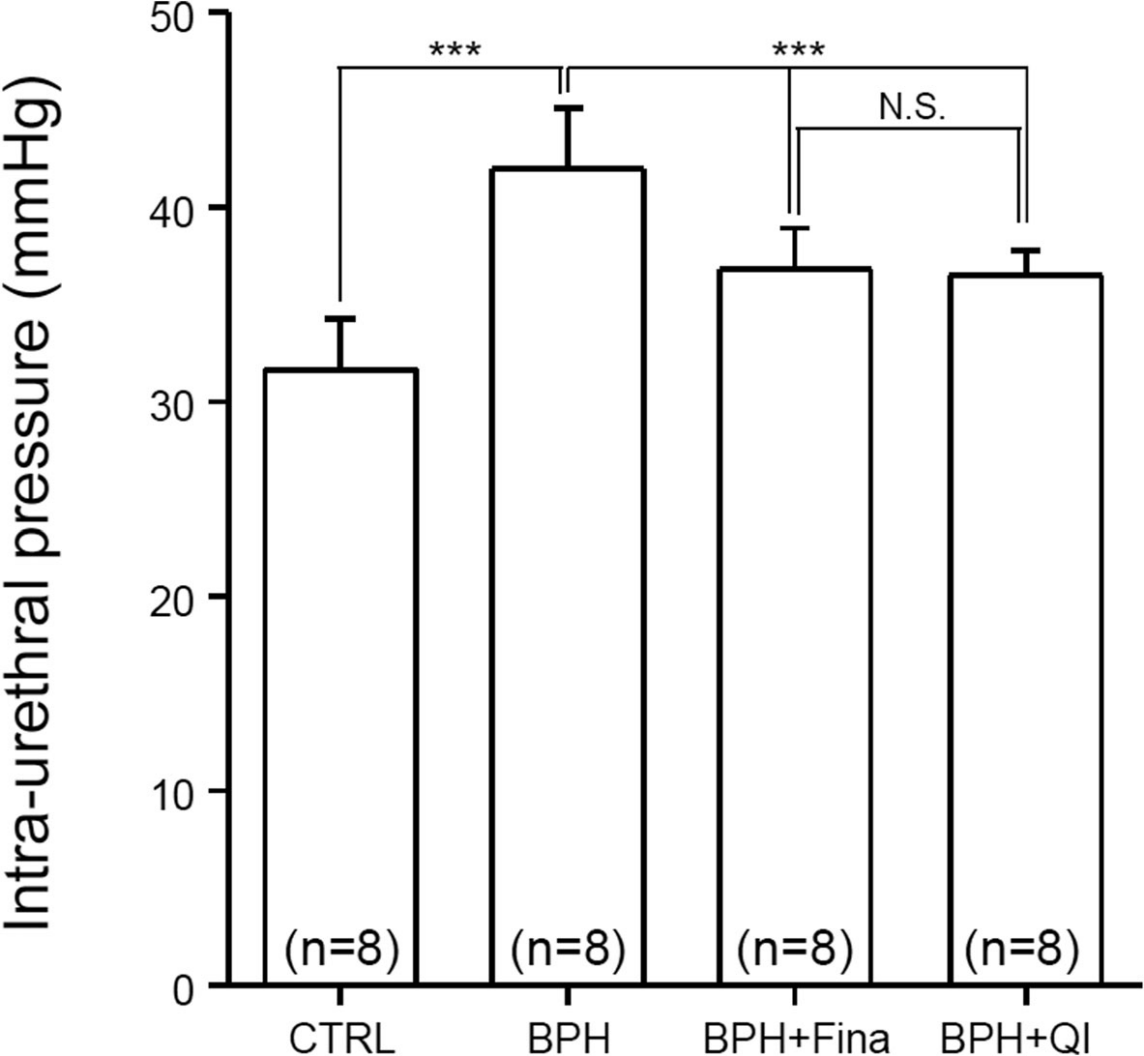
Effects of QI extract and Finasteride on basal IUP in anesthetized BPH-induced model rats. The basal IUP is derived from an average of 2 min (1 Hz sampling) of the first point of equilibrium. CTRL, Vehicle-treated animal; BPH, BPH-induced and non-treated animal; BPH + Fina, BPH induced and Finasteride (10 mg/kg/day, Oral) treated animals; BPH + QI, BPH induced and QI (150 mg/kg/day, Oral) - treated animals. Column and bars represent the mean and SEM (***P < 0.001)

The effect of BPH induction and QI administration on baseline IUP was measured (Fig. 1). Baseline IUP for the BPH-induced group (BPH) was 41.99 ± 3.11 mmHg, which was higher than 31.63 ± 2.61 mmHg measured for the control group. However, in the BP- induced group treated with Fina, there was a tendency for was 36.80 ± 2.12 mmHg (*n* = 8, *P* value< 0.05). IUP was 36.45 ± 1.30 mmHg in the BPH-induced group treated with QI, which was similar to the Fina group. The QI and Fina groups showed similar inhibition of BPH-induced elevation of urethral pressure.

### Effects of Phe injection on IUP changes

**Fig. 2:**
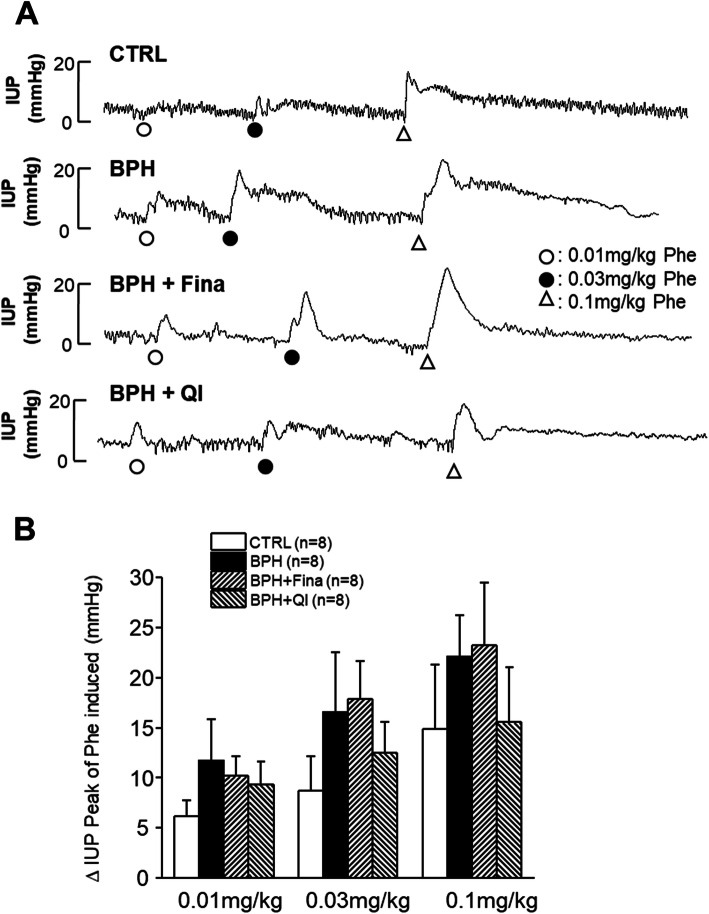
Effects of QI extract and Finasteride on electrostimulation (5 V, 5, 10, 20 Hz) induced an increase in IUP in anesthetized BPH-induced model rats. Urethra and surrounding prostate tissue were stimulated by a pair of fine platinum subdermal needles. CTRL, Vehicle-treated animal; BPH, BPH-induced and non-treated animal; BPH + Fina, BPH-induced and Finasteride (10 mg/kg/day, Oral)-treated animals; BPH + QI, BPH induced and QI (150 mg/kg/day, Oral)-treated animals. Each column and bars represent the mean and SEM (*P < 0.05)

We examined the changes in IUP for alpha-adrenergic stimulation induced by Phe administration in each group. In the 0.01 mg/kg Phe administered group, the peak values of IUP were 6.19 ± 1.55 mmHg, 11.67 ± 4.14 mmHg, 10.16 ± 1.99 mmHg, and 9.31 ± 2.31 mmHg for CTRL, BPH, Fina, and QI groups respectively. In the 0.03 mg/kg Phe administrated group, peak values of IUP were 8.74 ± 3.42 mmHg, 16.53 ± 5.98 mmHg, 17.89 ± 3.79 mmHg and 12.52 ± 3.06 mmHg for CTRL, BPH, Fina and QI groups respectively. In the case of 0.1 mg/kg Phe administration, 14.89 ± 6.39 mmHg, 22.09 ± 4.08 mmHg, 23.20 ± 6.26 mmHg and 15.53 ± 5.51 mmHg peak values of IUP were recorded for CTRL, BPH, Fina and QI groups respectively (Fig. 2b). The concentration of Phe injected was directly proportional to IUP. The increment in the IPU of the QI-treated group was significantly reduced compared to the increment in the Fina group.

### Effects of EFS on IUP changes

**Fig. 3:**
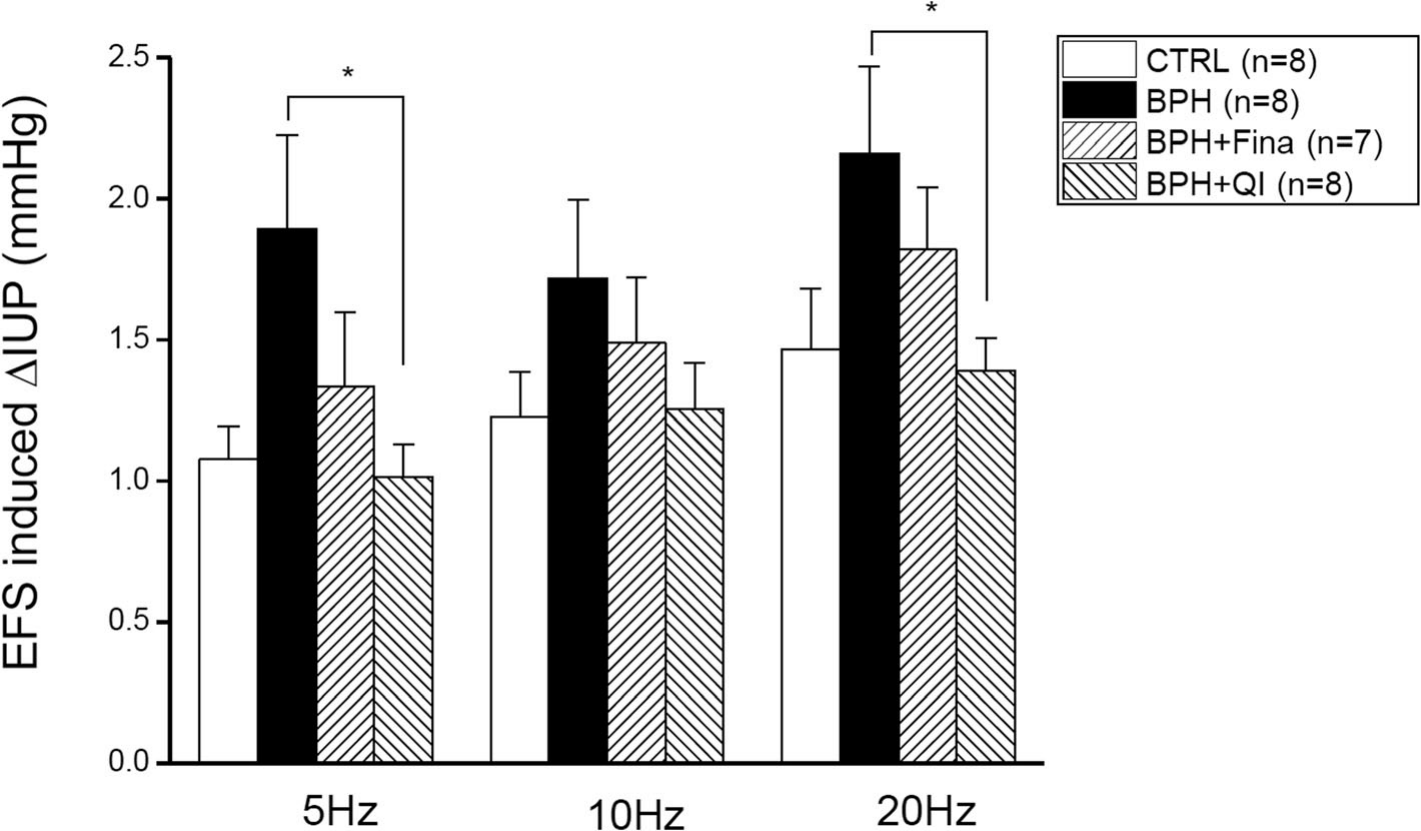
Effects of QI extract and Finasteride on intraurethral pressure increase peak and average value induced by acute Phe injection in anesthetized BPH-induced model rats. **a** Representative traces of each control and treatment group. **b** The intraurethral pressure increases the peak value induced by Phe (0.01, 0.03, 0.1 mg/kg IV). CTRL, Vehicle-treated animal; BPH, BPH-induced and non-treated animal; BPH + Fina, BPH induced and Finasteride (10 mg/kg/day, Oral) treated animals; BPH + QI, BPH induced and QI (150 mg/kg/day, Oral)-treated animals. Each column and bars represent the mean and SEM

We also examined the effect of hypogastric nerve stimulation on IUP in each group (Fig. 3). For stimulation at 5 Hz, the values for the CTRL, BPH, Fina, and QI groups were 1.08 ± 0.12 mmHg, 1.98 ± 0.33 mmHg, 1.33 ± 0.27 mmHg and 1.01 ± 0.11 mmHg, respectively. For the stimulation at 10 Hz, the values were 1.22 ± 0.16 mmHg, 1.72 ± 0.28 mmHg, 1.49 ± 0.23 mmHg and 1.25 ± 0.17 mmHg for CTRL, BPH, Fina, and QI respectively. At 20 Hz, 1.47 ± 0.21 mmHg, 2.16 ± 0.31 mmHg, 1.82 ± 0.22 mmHg and 1.38 ± 0.12 mmHg were measured for CTRL, BPH, Fina and QI respectively. Compared to CTRL, the response of the BPH group to the stimulation of 5 Hz EFS was significantly increased (*P* < 0.05). The Fina and QI groups, however, still showed a similar increase compared to CTRL.

### Effects of Fina and QI on BP

**Fig. 4:**
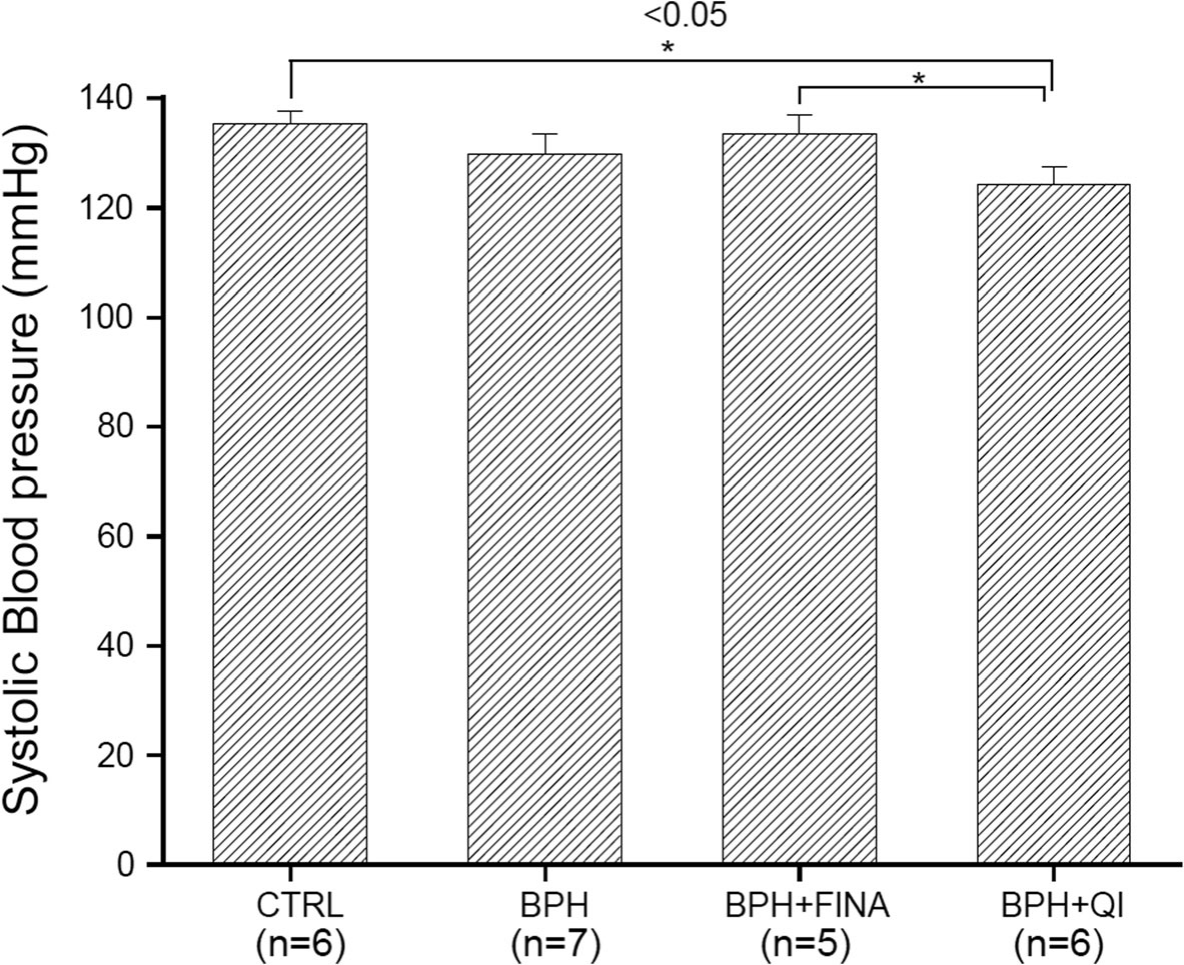
Effects of QI extract and Finasteride on systolic BP in anesthetized BPH-induced model rats. CTRL, Vehicle-treated animal; BPH, BPH-induced and non-treated animal; BPH + Fina, BPH induced and Finasteride (10 mg/kg/day, Oral) treated animals; BPH + QI, BPH induced and QI (150 mg/kg/day, Oral)-treated animals. Each column and bars represent the mean and SEM (*P < 0.05)

We evaluated changes in blood pressure in each group. The systolic BP of the CTRL, BPH, Fina, and QI groups were 135.40 ± 2.16 mmHg, 129.86 ± 3.58 mmHg, 133.54 ± 3.49 mmHg, and 124.33 ± 3.20 mmHg respectively (Fig. 4). There were no significant statistical differences between the CTRL, BPH, and Fina groups, but the QI group showed a significant decrease in systolic BP (*P* < 0.05). Therefore, QI has no adverse effects on the cardiovascular system.

### Effect of QI on the weight of male reproductive organs

**Fig. 5:**
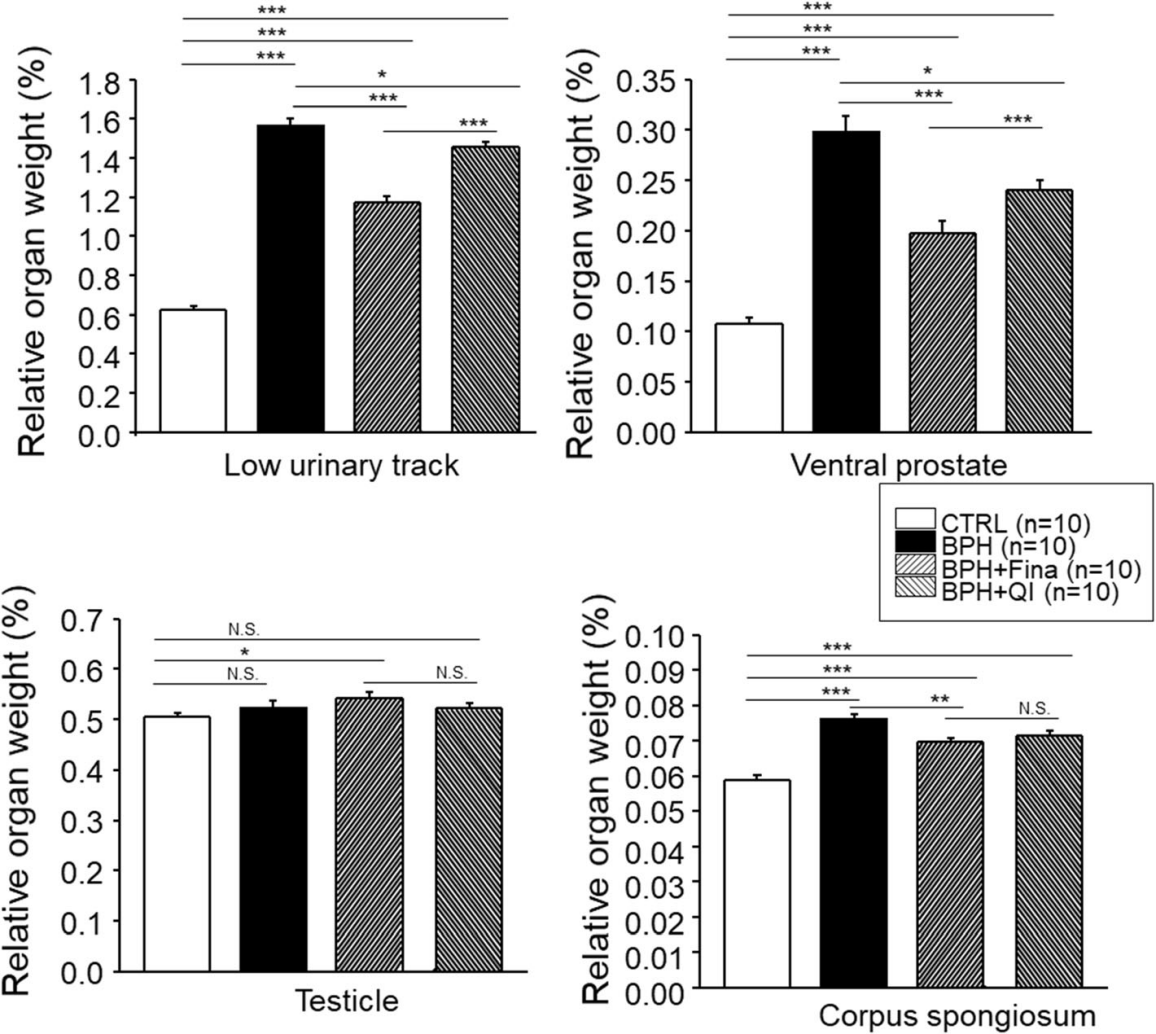
Effects of QI extract and Finasteride on the weight of various organs in control and treatment groups. The ratio of relative organ weight **a** low urinary tract, **b** ventral prostate, **c** testicular, **d** corpus spongiosum. CTRL, Vehicle-treated animal; BPH, BPH-induced and non-treated animal; BPH + Fina, BPH induced and Finasteride (10 mg/kg/day, Oral) treated animals; BPH + QI, BPH induced and QI (150 mg/kg/day, Oral)-treated animals. Each column and bars represent the mean and SEM (*P < 0.05, ** *P* < 0.01, ***P < 0.001)

We examined the ratio of LUT, VP, testicle, and CS for each organ relative to body weight. CTRL, BPH, Fina, and QI recorded LUT values of 0.624 ± 0.020%, 1.563 ± 0.026%, 1.176 ± 0.031%, and 1.457 ± 0.028% respectively (Fig. 5a). LUT weight was significantly decreased in the Fina group, but increased in the BPH group (*P* < 0.001). The QI groups also recorded decreased LUT weight, but it was less statistically significant (*P* < 0.05). Finely dissected VP weights were measured, and the values were 0.108 ± 0.006%, 0.298 ± 0.015%, 0.198 ± 0.012%, and 0.240 ± 0.009% for CTRL, BPH, Fina, and QI respectively, (Fig. 5b). The weight of VP of the Fina group was significantly lower compared to the BPH group (*p* < 0.001). The weight of VP in the QI group was significantly decreased (*P* < 0.05). The weights of testicles in CTRL, BPH, Fina, and QI groups were 0.504 ± 0.009%, 0.524 ± 0.014%, 0.542 ± 0.012%, and 0.521 ± 0.012%, respectively (Fig. 5c). There were no significant differences in testicle weight between the treated groups. The weights of CS were 0.059 ± 0.001%, 0.076 ± 0.001%, 0.070 ± 0.001%, and 0.072 ± 0.001% for CTRL, BPH, Fina, and QI, respectively (Fig. 5d). No significant difference was found between the Fina and QI groups, But the weight was greater in CS than in the CTRL group (*P* < 0.001).

### Effects of QI on serum CRP concentration

**Fig. 6:**
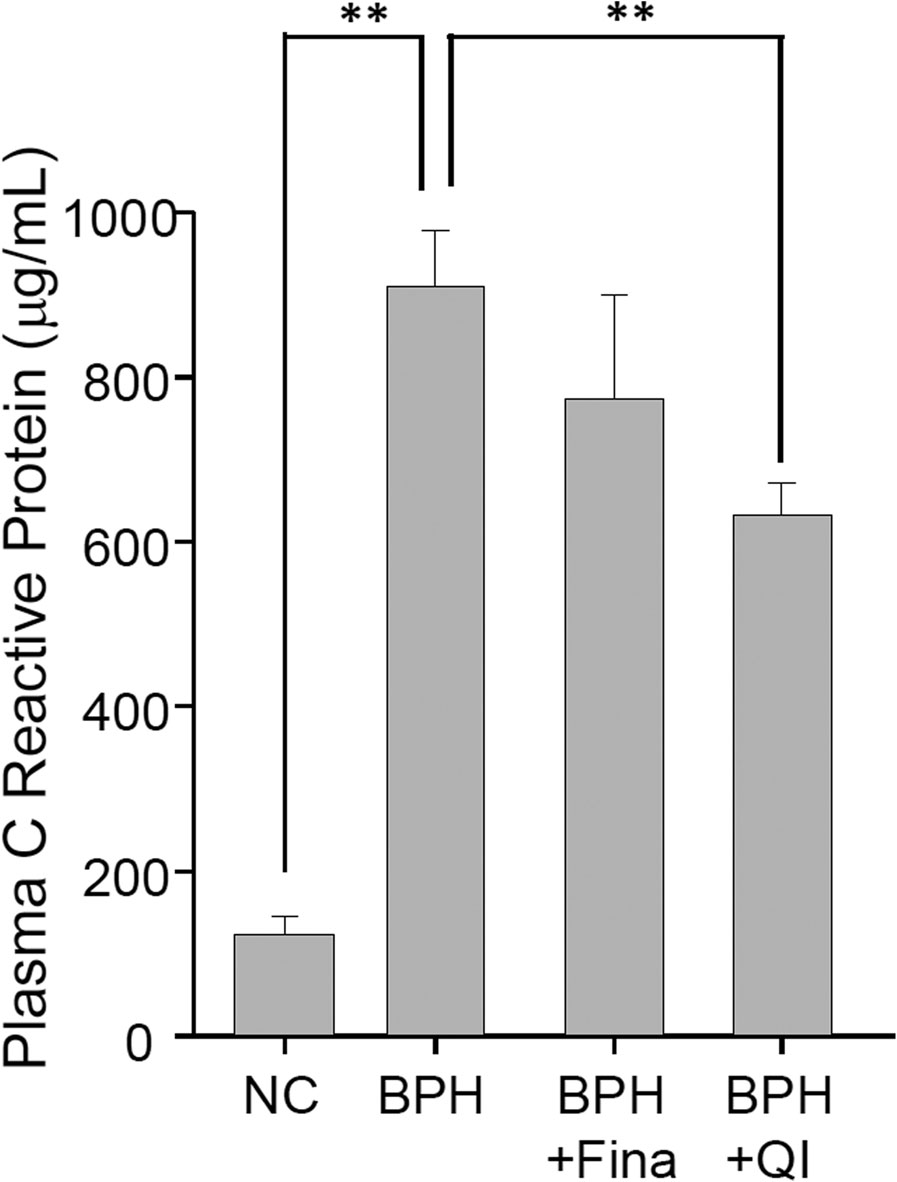
Effects of QI on serum C-reactive protein (CRP) concentration in serum. CTRL, Vehicle-treated animal; BPH, BPH-induced and non-treated animal; BPH + Fina, BPH induced and Finasteride (10 mg/kg/day, Oral) treated animals; BPH + QI, BPH induced and QI (150 mg/kg/day, Oral)-treated animals. Each column and vertical bar represents the mean and SEM (** *P* < 0.01)

The concentration of serum CRP was measured. Serum CRP concentration was significantly higher in the BPH (*P* < 0.05) than in the control group (Fig. 6). Serum CRP concentration was not significantly different in the Fina and BPH groups. The CRP concentrations in the QI groups were significantly lower compared to the BPH group (*P* < 0.05). The concentrations were 124.10 ± 21.87, 909.83 ± 67.91, 773.79 ± 127.15, and 632.63 ± 38.98 μg/mL for the CTRL, BPH, Fina, and QI groups, respectively (Fig. 6).

**Fig. 7:**
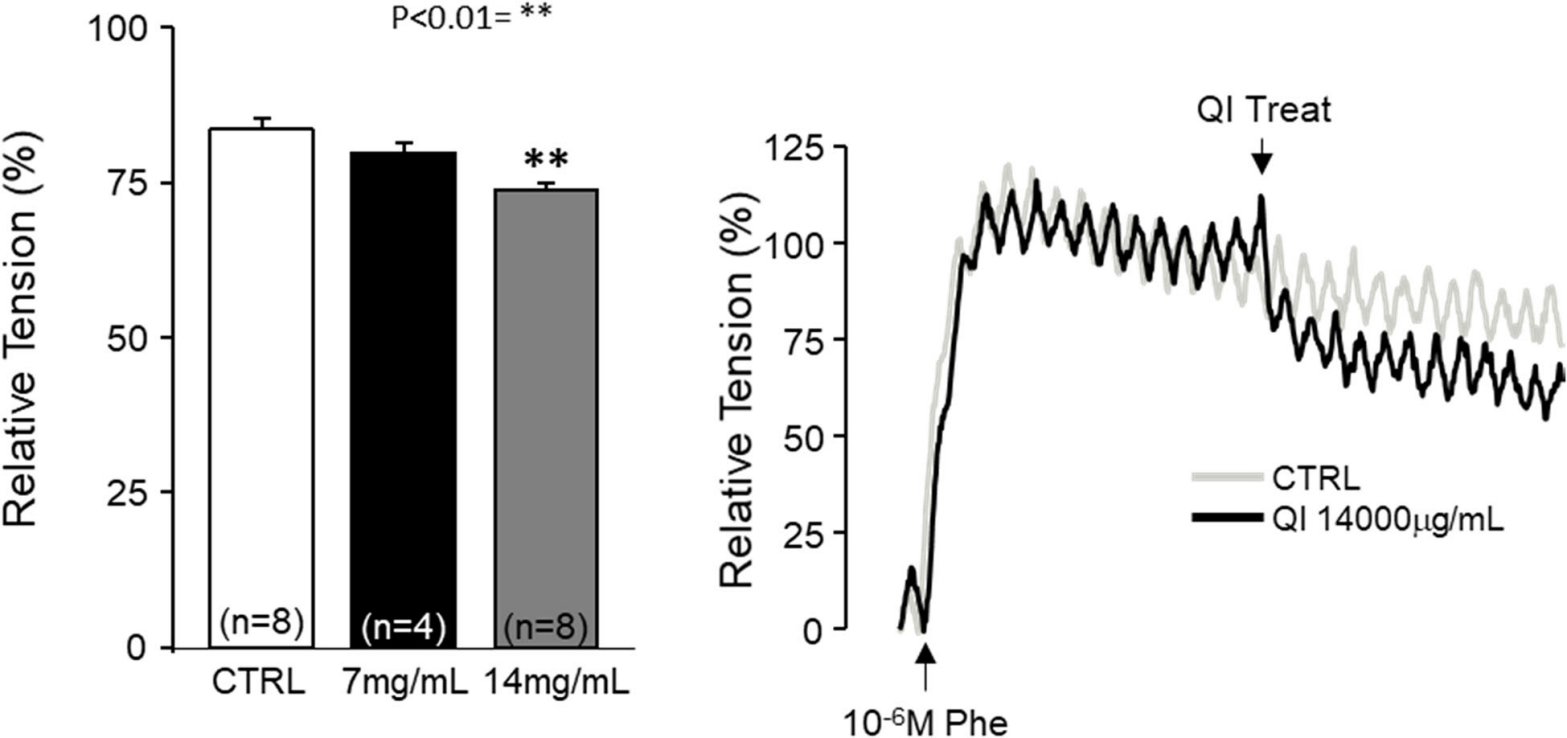
Effects of QI extract on phenylephrine precontracted ventral prostate strips **a**), control vehicle (CTRL), 7 mg/mL, and 14 mg/mL QI extracts were treated in the rat ventral prostate strip pre-contracted with 1 μM Phe. **b** Representative trace of effects of QI extract on Phe induced contraction on the ventral prostate strip. Each column and vertical bar represents the mean and SEM (**P < 0.01)

### Effects of QI on ventral prostate smooth muscle strip

We examined the effect of QI extract on Phe-induced contraction of the ventral prostate smooth muscle strip to test immediate action on prostate relaxation and its contribution to IUP release. Vehicle (DMSO); 7 mg/mL/mL, and 14 mg/mL of QI extract were added in the precontracted ventral prostate smooth muscle strip with 1 μM Phe. In the CTRL group, 83.78 ± 1.68% of maximal contraction remained after 5 min of treatment. Treatment with 7 mg/mL and 14 mg/mL QI reduced this to 79.61 ± 1.79% and 73.74 ± 1.09% respectively (*n* = 5–8, *p* < 0.01). Therefore, QI extract can inhibit Phe-induced contraction of ventral prostate smooth muscle in high concentrations (Fig. 7).

## Discussion

The prostate surrounds the neck of the bladder and urethra, and plays an important role in the male reproductive system [13]. Enlargement of the prostate can cause lower urinary tract symptoms such as urinary tract obstruction, urinary tract infection, bladder function depression, and erectile dysfunction due to the proliferation of the prostate tissue and compression of the urethra [14].

A recent study by Ub Wijerathne et al. demonstrated that QI reduced the epithelial hyperplasia of the prostate, blood testosterone, DHT in prostate tissue, and 5α-reductase [9]. Baek et al. also proposed the attenuation of prostatic hyperplasia via possible interactions of QI with α1 adrenergic receptor and androgen receptor [15]. These two studies evaluated the mechanisms of action of QI, but we confirmed in our study that QI could mitigate the increase in urethral pressure, which is the biggest practical symptom of enlarged prostate at effective doses in BPH rat models. The basal urethral pressure, adrenergic stimulated-elevation, and hypogastric nerve stimulated-elevation of intraurethral pressure were reduced in the QI-treated animals. We also confirmed that QI can reduce systemic CRP concentration. Systolic blood pressure, however, was not significantly reduced.

Dihydrotestosterone (DHT) increases in the prostate, although serum testosterone levels decrease with age. It has been reported that the activity of 5α-reductase, which converts testosterone to DHT, was increased in BPH [16]. Therefore, 5α-reductase inhibitors, such as finasteride or dutasteride, have been widely used in treating BPH [17]. Previously, Wijerathene et al. showed that QI extract inhibited 5α reductase type 2, cyclin D1, and caspase-3. Therefore, it is not surprising that QI extract reduced intraurethral pressure to ameliorate low urinary tract symptoms. As expected, QI treatment reduced the whole lower urinary tract as well as the ventral prostate in the relative organ weight, and intraurethral pressure. Although the rat prostate cannot surround the urethra as observed in humans, dorsal and ventral prostate enlargement can cause incomplete urinary voiding and increase the frequency of micturition [18].

QI extract alleviated intraurethral pressure in response to Phe administration compared to the BPH group, and IUP increased similarly in the QI and control groups as Phe concentration was increased in the QI group. This suggests that the administration of QI inhibits α adrenergic receptors in the prostate in vivo*,* while isolated ventral prostatic smooth muscle strips were only relaxed in high concentrations of QI treatment. The use of conventional α1-adrenoceptor antagonists that are structurally related (e.g., tamsulosin and indoramin) to prazosin could be limited by drug-related adverse cardiovascular effects, such as orthostatic hypotension [19]. The low selectivity of the receptor subtypes of α adrenergic receptor inhibitors can lead to dizziness due to vasodilation [20]. However, QI treatment did not affect BP. The systolic blood pressure of the QI group was lower than that of the CTRL group, and this is thought to be due to the antagonistic effects on androgen receptors. This suggests that QI mitigates the symptoms of BPH without side effects on the cardiovascular system.

## Conclusions

Alpha-blockers are believed to improve symptoms and flow rate by inducing relaxation of the smooth muscle neck and prostate area (dynamic component), while 5α-reductase inhibitors are believed to improve symptoms and flow rate by shrinking the transition zone of the prostate through hormonal mechanisms (static component). Therefore, combined therapy using α1 adrenergic blocker and 5α-reductase inhibitor may be more effective in relieving and preventing the progression of symptoms [21]. QI administration can result in symptomatic improvement in BPH. The mechanism of action of QI may be via inhibition of the 5α reducing activity with mild inhibition of α1 adrenergic receptor at the systemic level. Therefore, QI extract can significantly improve symptoms of BPH with fewer adverse effects and may also be used as a dietary supplement for prostate health.

## Data Availability

The data that support the findings of this study are available on request from the corresponding author on reasonable request.

## Abbreviations

BPH: Benign prostate hyperplasia
QI: *Quisqualis indica linn*
TP: Testosterone propionate
IUP: Intraurethral pressure
Phe: Phenylephrine
Fina: Finasteride
LUT: Which low urinary tract
VP: Ventral prostate
CS: Corpus spongiosum
DHT: Dihydrotestosterone
CRP: C-reactive protein
DMSO: Dimethyl sulfoxide
